# Reposition: Focalizing β-Alanine Metabolism and the Anti-Inflammatory Effects of Its Metabolite Based on Multi-Omics Datasets

**DOI:** 10.3390/ijms251910252

**Published:** 2024-09-24

**Authors:** Wenjun Luo, Haijun Zhang, Hao Zhang, Yixi Xu, Xiao Liu, Shijun Xu, Ping Wang

**Affiliations:** 1 State Key Laboratory of Southwestern Chinese Medicine Resources, Chengdu University of Traditional Chinese Medicine, Chengdu 610075, China; wenjunluoller@stu.cdutcm.edu.cn (W.L.); zhanghj@stu.cdutcm.edu.cn (H.Z.); haozhang@stu.cdutcm.edu.cn (H.Z.); wjxuyixi@stu.cdutcm.edu.cn (Y.X.); xushijun@cdutcm.edu.cn (S.X.); 2 School of Pharmacy, Chengdu University of Traditional Chinese Medicine, Chengdu 610075, China; 3 Institute of Material Medica Integration and Transformation for Brain Disorders, Chengdu University of Traditional Chinese Medicine, Chengdu 610075, China

**Keywords:** inflammation, multi-omics datasets, RAW264.7 cells, integration analysis, β-alanine metabolism

## Abstract

The incorporation of multi-omics data methodologies facilitates the concurrent examination of proteins, metabolites, and genes associated with inflammation, thereby leveraging multi-dimensional biological data to achieve a comprehensive understanding of the complexities involved in the progression of inflammation. Inspired by ensemble learning principles, we implemented ID normalization preprocessing, categorical sampling homogenization, and pathway enrichment across each sample matrix derived from multi-omics datasets available in the literature, directing our focus on inflammation-related targets within lipopolysaccharide (LPS)-stimulated RAW264.7 cells towards β-alanine metabolism. Additionally, through the use of LPS-treated RAW264.7 cells, we tentatively validated the anti-inflammatory properties of the metabolite Ureidopropionic acid, originating from β-alanine metabolism, by evaluating cell viability, nitric oxide production levels, and mRNA expression of inflammatory biomarkers. In conclusion, our research represents the first instance of an integrated analysis of multi-omics datasets pertaining to LPS-stimulated RAW264.7 cells as documented in the literature, underscoring the pivotal role of β-alanine metabolism in cellular inflammation and successfully identifying Ureidopropionic acid as a novel anti-inflammatory compound. Moreover, the findings from database predictions and molecular docking studies indicated that the inflammatory-related pathways and proteins may serve as potential mechanistic targets for Ureidopropionic acid.

## 1. Introduction

The swift evolution of contemporary high-throughput omics measurement technologies has established clustering analysis of multi-omics data, employing predictive algorithms derived from machine learning, as a significant area of interest in current systems biology research [1]. From both statistical and biological perspectives, the amalgamation of multi-dimensional datasets is expected to yield more advantageous results compared to analyses performed on single-layer data. The integration of multi-omics data enables the extraction of insights from diverse biological molecular layers, thereby presenting a promising pathway for a systematic and holistic comprehension of complex biological phenomena [2,3]. Common approaches for multi-omics integration include mathematical matrix factorization, artificial intelligence-based neural network models, Bayesian statistics, and metric learning [1]. The intrinsic heterogeneity of multi-omics data necessitates the implementation of effective preprocessing techniques, such as feature selection, filtering, and standardization [4]. A range of bioinformatics tools and data management systems offer substantial support for the clustering of multi-omics data, facilitating the identification of potential core disease targets based on analytical results and the exploration of therapeutic interventions with potential druggability.

The inflammatory response system is characterized as a complex and dynamic process that functions across multiple molecular levels, with its regulation dependent on gene expression, protein synthesis, and metabolite production. These elements collectively contribute to intricate relationships within molecular biology, including the transcriptional regulation between genes and proteins. Metabolites play dual roles as both substrates and products in enzymatic reactions, as well as precursors for the synthesis of proteins and RNAs. They variably modulate the activities of enzymes or transcription factors, thereby indirectly affecting the expression of multiple target genes [5]. Consequently, a comprehensive investigation and understanding of the initiation and progression of inflammation require a multilayered and multi-perspective approach, fully utilizing multi-omics technology to provide novel insights or methodologies for elucidating inflammatory diseases.

Recent investigations into inflammatory mechanisms utilizing omics technologies have produced significant findings. The changes in post-translational modifications (PTMs) during macrophage inflammatory responses have been thoroughly characterized through various omics techniques, including proteomics, phosphorylation modification, acetylation modification, and ubiquitination modification [6]. Detailed analyses have resulted in the identification of key signaling pathways and novel drug targets by constructing a systematic map of multi-omics data at different stages of silicosis [7]. Additionally, the role of mitochondrial complex I in microglia has been identified as a promoter of neuroinflammation through the maintenance of microglial activation, as evidenced by multi-omics approaches [8]. Nevertheless, there remains a scarcity of reports addressing the underlying mechanisms of cellular inflammation through clustering analysis of multi-omics data across various dimensions.

In this study, we employed an ensemble learning approach to integrate and cluster relevant omics datasets obtained from the literature concerning LPS-stimulated RAW264.7 mouse macrophages. Time-resolved omics datasets, which consist of data collected at various time points [9], are frequently utilized to construct novel biological networks and to clarify the interrelationships among omics networks in response to specific biological stimuli [5]. Consequently, the time-dependent response sequences across different omics were utilized as classification output conditions to generate multiple time-resolved multi-omics correlation maps, effectively depicting the upstream and downstream interconnections among diverse omics data. Following the enrichment and classification of these maps, the conditional output revealed either interconnected or distinct pathways. Previously neglected pathways and targets from our existing database were re-assessed, and their potential associations with the inflammatory response were predicted. Ultimately, the anti-inflammatory effects of the metabolite Ureidopropionic acid, which is derived from β-alanine metabolism, were evaluated in vitro [10,11].

## 2. Results

### 2.1. Presentation of Multi-Omics Data from the Literature

**Figure 1:**
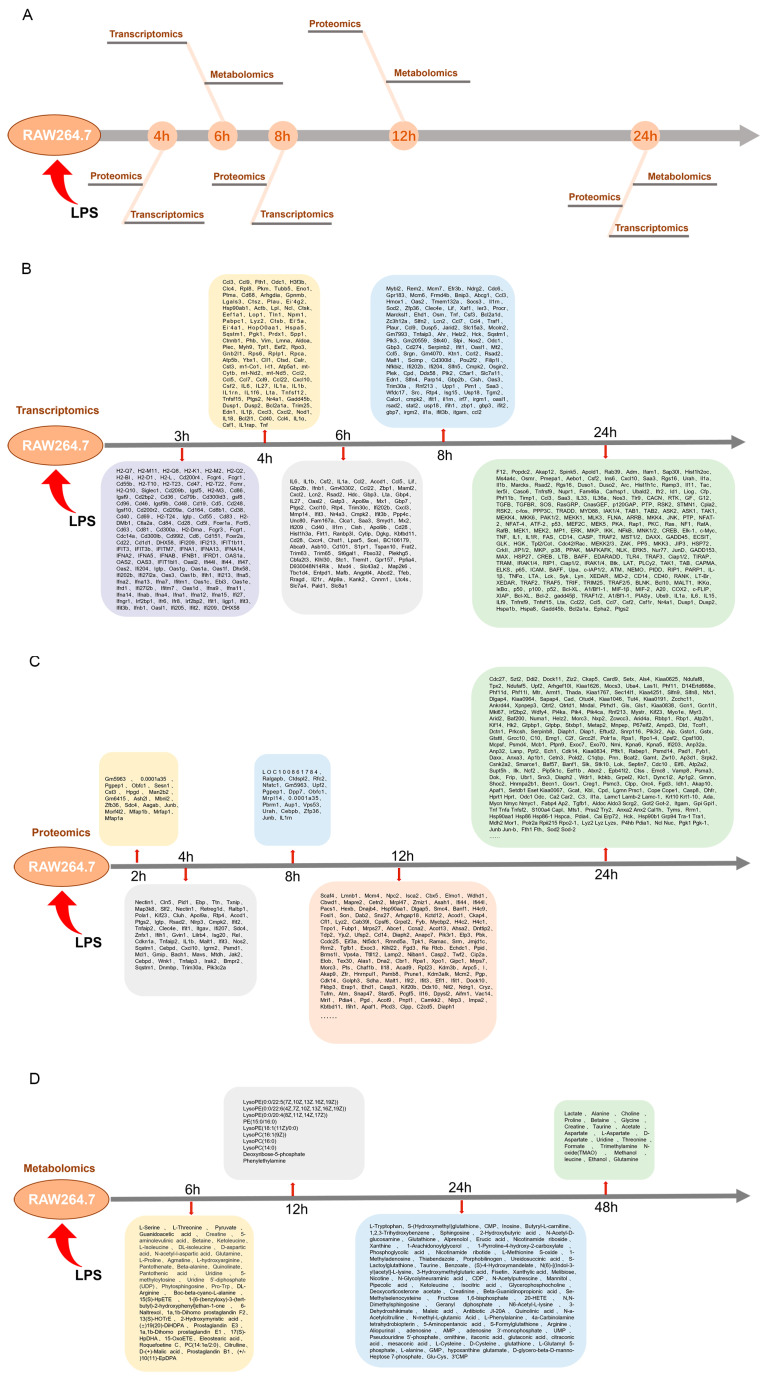
(**A**) Cross-integrated presentation of the data of each group under temporal resolution. (**B**) Transcriptomics section of differentially expressed genes. (**C**) Proteomics section of differentially expressed proteins. (**D**) Metabolomics section of differentially expressed metabolites.

The search terms utilized included ‘RAW264.7, LPS, transcriptomics’, ‘RAW264.7, LPS, proteomics’, and ‘RAW264.7, LPS, metabolomics’. The literature review encompassed publications from 1 January 2014 to 1 January 2024. The data sampling time points for the primary histological samples were predominantly focused on 4, 6, 8, 12, and 24 h (see Figure 1A). Each piece of literature was meticulously examined for its findings in transcriptomics [12,13,14,15,16,17,18,19,20,21,22,23,24], proteomics [25,26,27,28,29], and metabolomics [30,31,32,33,34,35], organized according to their respective sampling time points (refer to Figure 1B–D) (Supplementary Materials S1–S3).

### 2.2. Conversion of Omics-Related Differential Gene Encoding

**Figure 2:**
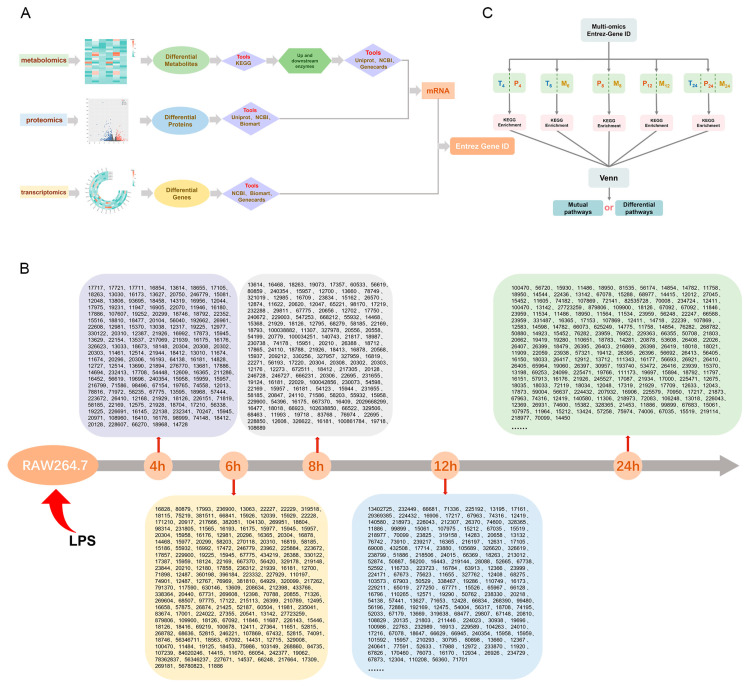
(**A**) Flowchart for normalized preprocessing of multi-omics data. (**B**) Entrez Gene ID partial arrangement of multi-omics data based on time resolution. (**C**) Preliminary construction of classification sampling model. T: Transcriptomics, P: Proteomics, M: Metabolomics. The sampling time is represented by a subscript number.

In light of the multi-dimensional hierarchical heterogeneity exhibited by various omics data, distinct pretreatment methodologies were employed for each type of omics information. Utilizing databases such as NCBI, Biomart, GeneCards, and UniProt (see Figure 2A), the data from each group were systematically screened and organized, ultimately being normalized to the Entrez Gene ID (see Figure 2B) (Supplementary Materials S5). This normalization provided a foundational dataset for the subsequent construction of algorithms for ensemble learning.

In contrast to genes and proteins, metabolites function as both substrates and products within enzymatic reactions. They serve as monomers for the synthesis of proteins and RNA and possess the ability to allosterically modulate the activity of enzymes or transcription factors, thereby indirectly influencing the expression of multiple genes. Consequently, in this study, we identified and cataloged the upstream and downstream catalytic enzymes associated with metabolites across all metabolic pathways as delineated in the KEGG database (Supplementary Materials S4). Subsequently, the genes corresponding to these catalytic enzymes were queried and integrated into the Entrez Gene ID framework.

A preliminary classification sampling model was constructed (see Figure 2C). Based on a temporal sequence, a total of five instances were selected, and follow-up analyses were conducted according to these five subsets.

### 2.3. Temporal Pathway Enrichment Analysis of Multi-Omics Data

**Figure 3:**
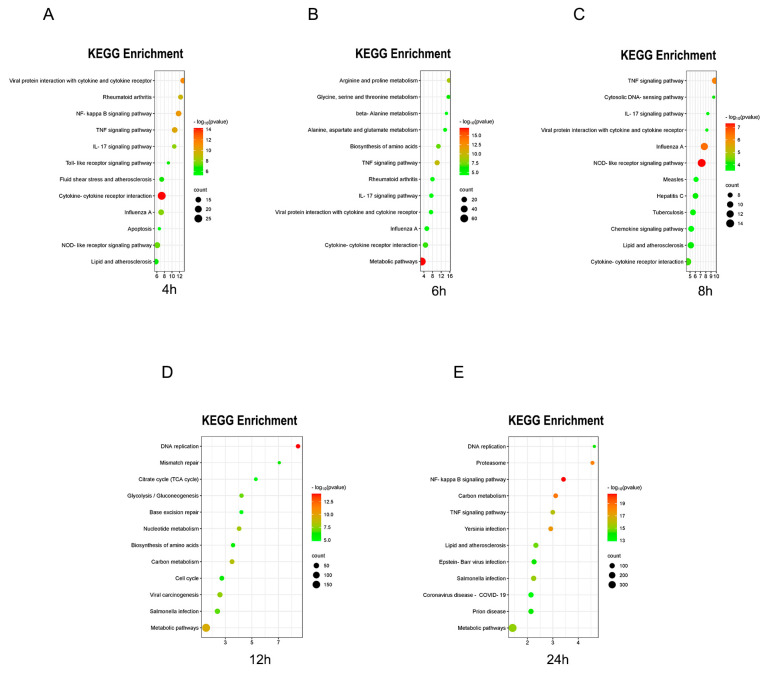

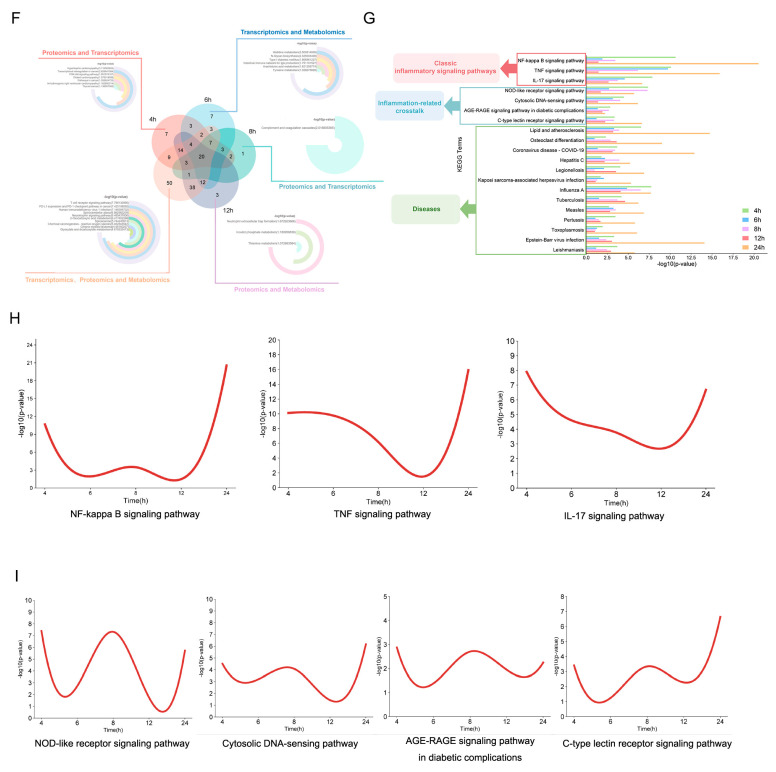
(**A**–**E**) KEGG pathway enrichment analysis diagrams at T4, T6, T8, T12, T24 (h). (**F**) Venn intersection analysis result diagram. (**G**) Comparison of enrichment significance at different time points under each mutual pathway. (**H**) Chronological chart about the expression of time-dependent signal dynamics oscillation mechanism of classical inflammatory pathway. (**I**) Chronological chart of the expression of time-dependent signal dynamics oscillation mechanism of inflammation-related signal crosstalk pathways.

The multi-omics data, represented by Entrez Gene IDs at each time point, were mapped to the DAVID database to conduct pathway enrichment analysis (Supplementary Materials S6). The top twelve pathways identified at each time point were selected for the construction of enrichment diagrams (Figure 3A–E). A Venn intersection analysis was performed on the enriched pathways across the various time points, revealing a total of 20 common pathways for further examination (Figure 3F) (Supplementary Materials S7). These pathways included classical signaling pathways associated with inflammation, disease pathways, and pathways related to the crosstalk of inflammation signals.

To assess the significance of the common pathways across different time points, a time series figure was generated (Figure 3G). Notably, the classical signaling pathways of inflammation and the crosstalk pathways exhibited a time-linear oscillation phenomenon, which aligns with the molecular mechanisms underlying the time-dependent signal dynamics observed in the LPS-stimulated RAW264.7 cell inflammation model (Figure 3H–I). This analysis supports the feasibility of employing an ensemble learning model to identify new targets for multi-omics clustering. Furthermore, it highlights the unique characteristics of this inflammatory system, characterized by rapid responses and significant dynamic changes consistent with the molecular mechanisms predicted by the validated iterative mathematical model.

The aforementioned common pathways reflect shared injury responses and are not specific to any single condition. However, through the application of various omics techniques for both qualitative and quantitative assessments of inflammatory indicators, these fundamental common damage indicators may serve as preliminary screening markers during the inflammatory response, thereby informing subsequent mechanistic investigations.

Additionally, each time point exhibited distinct differential enrichment pathways (Figure 3F). This raises the hypothesis that novel targets within these differential pathways may be implicated in the regulation of inflammation-related processes.

### 2.4. Repositioning of Core Targets within the Multi-Omics Time-Difference Signaling Pathway and Identification of Potential Intervention Agents

The findings from the Venn intersection analysis indicated the presence of distinct pathways at each time point (Figure 3F). At various modeling intervals, certain inflammatory biomarkers and pathways elicited by the LPS-stimulated inflammatory model exhibited specificity. In our investigation of differential pathways at each time point, we concentrated on the early inflammatory phase, repositioning the core targets of multi-omics related to cellular inflammation within specific pathways and examining potential intervention agents.

**Figure 4:**
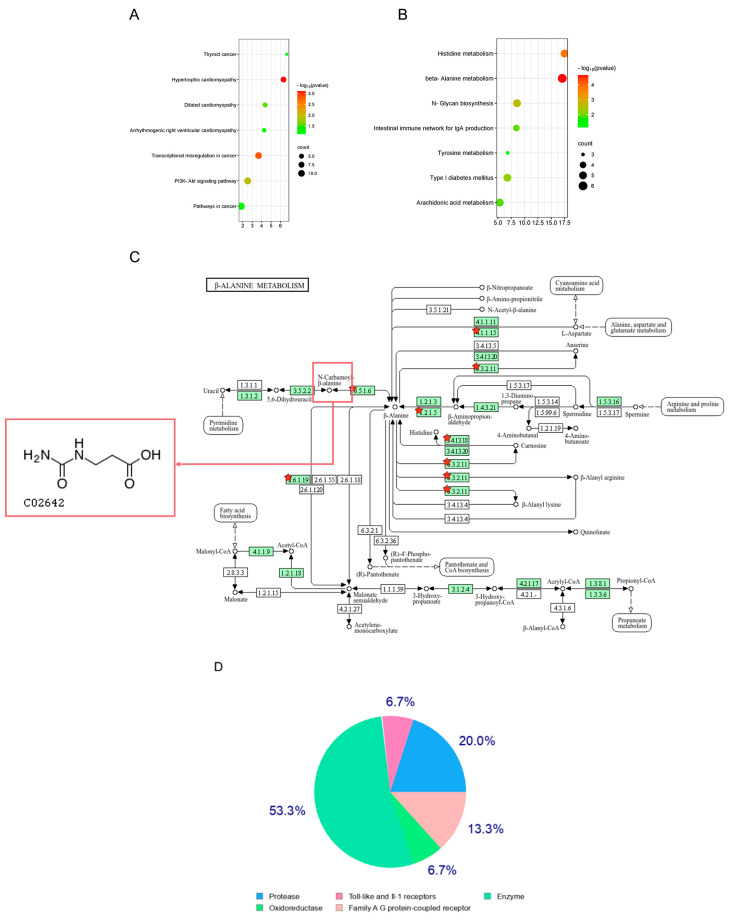
(**A**) LPS stimulated RAW264.7 cells for 4 h to specifically enrich the signal pathway diagram. (**B**) LPS stimulated RAW264.7 cells for 6 h to specifically enrich the signal pathway diagram. (**C**) The annotation map of potential targets of β-alanine metabolism and the structural formula of compound Ureidopropionic acid, starts represented differential genes *enriched in the pathway*. (**D**) SwissTargetPrediction database predicted the target chart of Ureidopropionic acid.

At the 4-h sampling time point (Figure 4A), the PI3K-AKT signaling pathway emerged as a significantly enriched pathway. This pathway is recognized as a transduction mechanism for signals associated with extracellular cell survival and growth [36,37]. The interplay between the PI3K-AKT pathway and inflammation is thought to involve the regulation of downstream NF-κB signaling through the phosphorylation of IKKα and Tpl2 [38]. Numerous studies have documented interventions targeting inflammation via the PI3K-AKT pathway [39,40,41].

At the 6 h sampling time point (Figure 4B), β-alanine metabolism was identified as the most significant pathway. Consequently, our initial focus shifted to β-alanine metabolism to investigate its potential core targets following repositioning.

In the annotation map of potential targets associated with β-alanine metabolism (Figure 4C), the differential genes *Upb1*, *Carns1*, *Abat*, *Gad1*, *Aldh3*, and *Cndp*, which was enriched within the metabolic pathway, were identified as substrates and products of catalytic enzymes. These may serve as intervention agents to modulate the inflammatory response mechanism. Notably, Upb1 functions as a catalytic enzyme involved in the synthesis of Ureidopropionic acid [42], while other catalytic enzymes implicated in the production of various compounds have been reported to have associations with inflammation in contemporary research [43,44,45,46,47,48,49]. Therefore, Ureidopropionic acid was selected as a candidate intervention agent to assess its role in the regulation of inflammation.

The SwissTargetPrediction database was utilized to predict the targets of Ureidopropionic acid (Figure 4D). The results indicated that the predicted targets related to Toll-like and IL-1 receptors constituted 6.7% of the total, suggesting that Ureidopropionic acid may play a role in the regulation of inflammation through these receptor targets.

### 2.5. Validation of the Anti-Inflammatory Effects of Ureidopropionic Acid

#### 2.5.1. Protective Effect of Ureidopropionic Acid on LPS-Stimulated RAW2674.7 Cells

**Figure 5:**
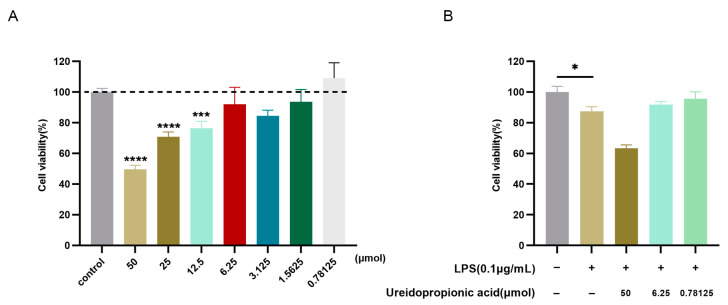
The cell viability of RAW264.7 cells was measured by a CCK-8 assay. (**A**) The effect of Ureidopropionic acid treatment for 24 h on the proliferation activity of RAW264.7 mouse macrophages. (**B**) The effect of Ureidopropionic acid on the proliferation activity of RAW264.7 cells treated with 0.1 μg/mL LPS. * *p* < 0.05, *** *p* < 0.001, **** *p* < 0.0001.

In this study, RAW264.7 cells were subjected to treatment with varying concentrations of Ureidopropionic acid to assess cytotoxicity. As illustrated in Figure 5A, the concentration range of Ureidopropionic acid that did not adversely affect the viability of RAW264.7 cells was identified as 0.78125 to 6.25 μmol, while concentrations ranging from 12.5 to 50 μmol exhibited a significant impact. This finding suggests that Ureidopropionic acid does not exert a notable inhibitory effect on the proliferation of RAW264.7 macrophages within the aforementioned concentration range.

Informed by differential enrichment pathway analyses of Ureidopropionic acid at the 6 h mark of LPS stimulation, as reported in existing literature, this study selected a 6 h LPS stimulation period for modeling purposes. As depicted in Figure 5B, a decrease in cell viability was observed following 6 h of LPS stimulation (*p* < 0.05). Conversely, the administration of Ureidopropionic acid at concentrations of 6.25 μmol and 0.78125 μmol for the same duration resulted in an increase in cell viability. Consequently, these two concentrations were chosen for further experimental investigations.

#### 2.5.2. Ureidopropionic Acid Attenuated the Expression of *IL-6* and *IL-1β* mRNA in LPS-Stimulated RAW264.7 Cells

**Figure 6:**
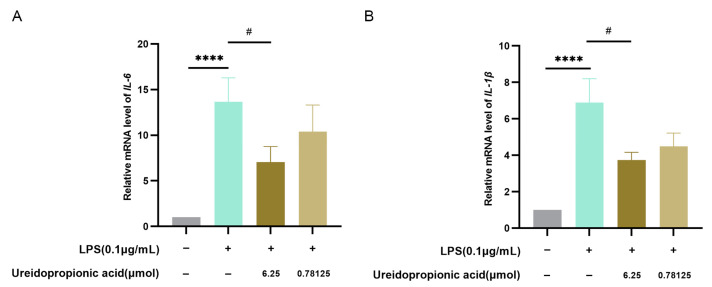
(**A**) The expression level of *IL-6* mRNA in RAW264.7 cells treated with each group was determined by qRT-PCR (*n* = 21). (**B**) The expression level of *IL-1β* mRNA in RAW264.7 cells treated with each group was determined by qRT-PCR (*n* = 24). ^#^ *p* < 0.05. **** *p* < 0.0001.

As illustrated in Figure 6A,B, treatment with Ureidopropionic acid at a concentration of 6.25 μmol resulted in a significant reduction in the expression levels of *IL-6* and *IL-1β* mRNA induced by LPS (*p* < 0.05).

#### 2.5.3. Ureidopropionic Acid Suppresses TNF-α mRNA Expression in LPS-Stimulated RAW264.7 Cells

**Figure 7:**
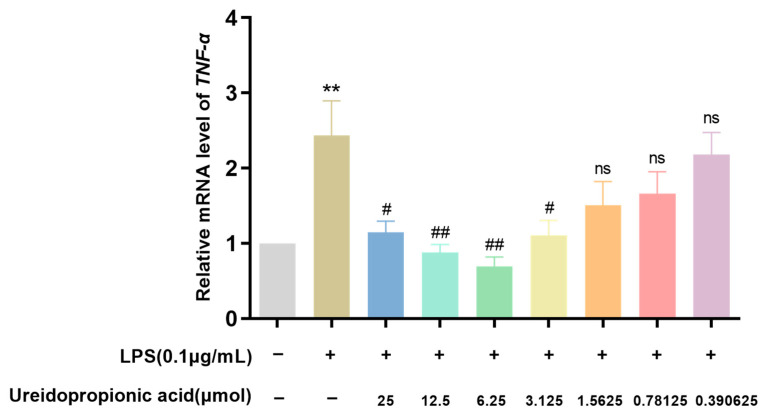
The expression level of *TNF-α* mRNA in RAW264.7 cells treated with each group was determined by qRT-PCR (*n* = 9). ^#^ *p* < 0.05. ^##^ *p* < 0.01. ** *p* < 0.01. ns *p* > 0.05.

As shown in Figure 7, in the 25, 12.5, 6.25, and 3.125 μmol groups, the administration of Ureidopropionic acid decreased the expression of *TNF-α* mRNA stimulated by LPS (*p* < 0.05).

As illustrated in Figure 7, the administration of Ureidopropionic acid at concentrations of 25, 12.5, 6.25, and 3.125 μmol resulted in a significant reduction in the expression of *TNF-α* mRNA in LPS-stimulated RAW264.7 cells (*p* < 0.05). The transcriptional regulation of TNF expression is primarily mediated by the MyD88-NF-κB signaling pathway. Previous studies have established that upon LPS stimulation, the mRNA expression of *TNF-α* in macrophages increases rapidly, peaking within a brief time frame, followed by a swift decline after one hour [50]. Consequently, in our assessment of relative *TNF-α* mRNA levels, we opted to shorten the modeling duration to 0.5 h, with sampling conducted within one hour. The findings indicate that Ureidopropionic acid can significantly modulate the transcriptional levels of *TNF-α* during the initial phase of inflammatory stimulation.

#### 2.5.4. Ureidopropionic Acid Inhibits LPS-Induced Nitric Oxide Synthesis in RAW264.7 Cells

**Figure 8:**
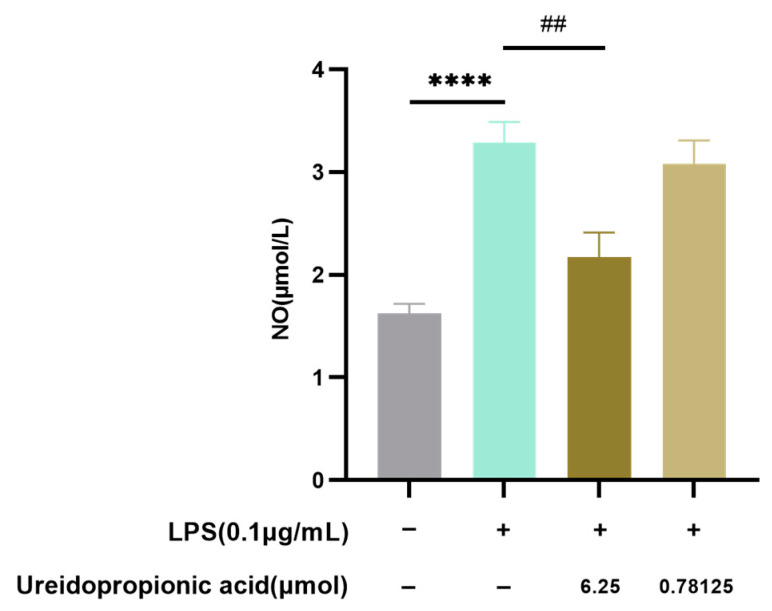
The level of NO in the supernatant of each group was determined by the Griess method (*n* = 18). ^##^ *p* < 0.01. **** *p* < 0.0001.

As demonstrated in Figure 8, the administration of Ureidopropionic acid led to a marked decrease in nitric oxide production triggered by LPS (*p* < 0.01).

#### 2.5.5. Molecular Docking of Ureidopropionic Acid with Autocatalytic Enzymes and Potential Targets

In the annotation map pertaining to the potential targets involved in β-alanine metabolism (Figure 4C), Upb1 was identified as the gene encoding β-ureidopropionase, an enzyme that functions as a hydrolase, facilitating the conversion of Ureidopropionic acid into β-alanine, ammonia, and carbon dioxide [51].

**Figure 9:**
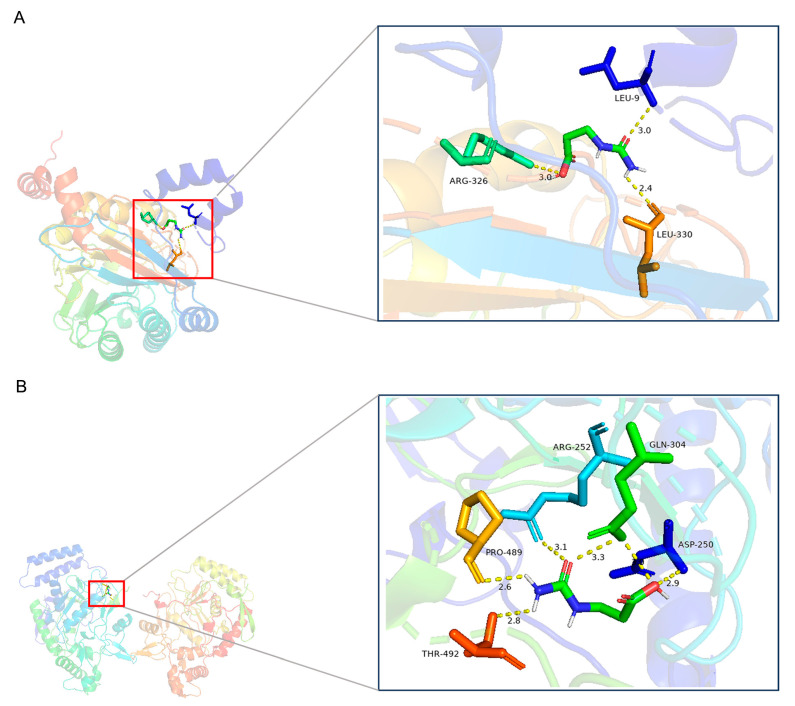
(**A**) Molecular docking diagram of Ureidopropionic acid and β-ureidopropionase by pymol 2.5 [52]. (**B**) Molecular docking diagram of Ureidopropionic acid and nitric oxide synthase by pymol 2.5 [52].

Initially, molecular docking studies were performed using the Autodock Vina software (autodock_vina_1_1_2) to assess the interactions between β-ureidopropionase and both Ureidopropionic acid and β-alanine. It is generally observed that the binding affinity of catalytic enzymes for their substrates exceeds that for their products. The results of the molecular docking analysis (Table 1) indicated binding energies of −5.1 kcal/mol for the interaction between Upb1-encoded β-ureidopropionase and Ureidopropionic acid, and −3.7 kcal/mol for the interaction with β-alanine. Subsequently, molecular docking of the *iNOS*-encoded protein, nitric oxide synthase, with Ureidopropionic acid was also conducted using the Autodock Vina software, yielding a binding energy of −5.3 kcal/mol. This finding suggests a strong affinity between nitric oxide synthase and the ligand molecule Ureidopropionic acid (Figure 9A,B). This target may be significantly associated with the mechanism by which Ureidopropionic acid modulates the inflammatory response.

## 3. Discussion

The dataset employed in this investigation was derived from multi-omics datasets that encompass a variety of feature modules documented in the existing literature. The integration or fusion of these datasets can significantly enhance the comprehension of sample variability. The concept of latent variables is instrumental in facilitating the integrated analysis of multiple datasets that share a common mode [53]. This study was inspired by the principles of ensemble learning, which led to the formulation of a classification sampling model. The implementation of this algorithm, alongside a series of bioinformatics analyses, revealed that the significance of enriched pathways within the inflammatory system of ‘LPS-stimulated RAW264.7 mouse macrophages’ could elucidate specific molecular mechanisms linked to time-dependent signaling dynamics, as both predicted and confirmed by iterative mathematical models.

In the context of literature data statistics, two predominant methodologies for addressing systematic bias are meta-analysis and data merging. Meta-analysis involves calculating relevant statistics for individual datasets before their aggregation, while data merging entails analyzing pooled data after bias removal. A comparative evaluation was conducted using both synthetic and real data, utilizing two manually curated microarray compendia [54]. Generally, algorithms and models associated with ensemble learning may overlook certain pathways or targets due to the reduced significance of constructing algorithmic models in isolation. However, low abundance or diminished significance does not inherently imply a lack of activity. Consequently, this study adopted the data merging approach, emphasizing the initial data from multidimensional histology following manual normalization preprocessing to delineate microarrays for each section. An integrated classification sampling model was subsequently reconstructed, with subsets categorized based on temporal discrimination to facilitate separate bioinformatics analyses of the responses. This methodology augmented both the diversity and quantity of genes identified in the original omics data, resulting in the identification of new pathways or an enhancement in the significance of certain pathways during the multi-omics enrichment analysis at each time point. Notably, β-alanine metabolism was initially overlooked as a significant pathway for anti-inflammatory target discovery due to its low significance and abundance within the individual omics network. However, following the model’s construction post-normalization preprocessing, the significance of β-alanine metabolism was markedly increased, and novel compounds with potential drug-forming properties within this metabolic pathway, specifically Ureidopropionic acid, were identified and preliminarily validated for their anti-inflammatory effects.

Macrophages play a crucial role in the inflammatory response, serving as a vital component of the innate immune system and engaging in a wide range of physiological and pathological processes. Their involvement in the development of various inflammatory diseases positions them as a focal point for inflammation research and therapeutic strategy development. The ‘LPS-stimulated RAW264.7 mouse macrophages’ system is a well-established in vitro model for investigating inflammatory responses. RAW264.7 cells exhibit rapid responses to LPS stimulation, characterized by significant dynamic changes governed by time-dependent signaling mechanisms. In this inflammatory context, inflammation-related signaling pathways reach peak activation within minutes to hours following LPS stimulation, resulting in the production and release of numerous pro-inflammatory factors and the establishment of a complex interaction network. The expression of key enzymes in metabolic pathways is modulated through transcriptional and post-translational modifications, thereby influencing metabolic pathways [55]. Thus, early intervention is critical in this inflammatory system, as it can effectively inhibit the signaling of relevant pathways and positively regulate associated metabolic pathways to mitigate the production of inflammatory mediators, control the inflammatory response, and provide optimal treatment within the therapeutic window to prevent irreversible damage.

In light of the fundamental principles of gene expression, there exists a discourse regarding whether different omics layers, such as the proteome and transcriptome, should be sampled at distinct time points to avoid the accumulation of noise resulting from temporal bias. It is essential to acknowledge that timing discrepancies exist not only between omics layers but also within the layers themselves [56]. For instance, in the metabolome, second messengers are synthesized rapidly within seconds to minutes [57], while energy metabolism undergoes changes over minutes to hours [58]. There is no universally optimal time point for each omics layer, nor is there an ideal time interval for sampling between different omics layers [56]. Throughout our investigative research, preprocessing was employed to eliminate duplicate terms, thereby mitigating the accumulation of noise in the comprehensive analysis. Consequently, we opted to integrate and normalize sample data from various omics layers at the same time point within this inflammatory model. Furthermore, our findings indicated that the temporal order of pathway significance in the shared signaling pathways, as determined through Venn intersection analysis, exhibited an oscillatory trend consistent with the oscillatory response of intra-pathway signaling dynamics. This observation validated the construction of our model and demonstrated the feasibility of our data integration and normalization approach.

Utilizing the SwissTargetPrediction database, we predicted the target of Ureidopropionic acid and found that it may be involved in inflammatory regulation by modulating Toll-like and IL-1 receptors. The activation of Toll-like receptors (TLRs) is closely associated with the initiation of inflammatory responses, which can activate various transcription factors that further promote the expression of inflammatory cytokines. Within the Toll-like receptor family, TLR4 directly recognizes lipopolysaccharide (LPS) stimulation and initiates a series of subsequent inflammatory responses. Upon binding to LPS, TLR4 is activated via the myeloid differentiation factor 88 (MyD88)-dependent pathway, leading to the activation of IL-1 receptor-associated kinases (IRAKs). The activated IRAKs facilitate the release and translocation of the NF-κB complex to the nucleus, where NF-κB binds to the promoter regions of *TNF-α*, *IL-6*, *IL-1β*, and other genes, thereby stimulating the transcription of *TNF-α*, *IL-6*, and *IL-1β* mRNA [59]. Similarly, when NF-κB is activated, it can bind directly to the promoter of the *iNOS* gene, promoting the transcription of *iNOS* mRNA and subsequently increasing the expression of *iNOS* proteins. *iNOS* catalyzes the production of nitric oxide (NO) and citrulline from L-arginine and oxygen. Under LPS-stimulated inflammatory conditions, elevated expression of *iNOS* results in increased NO production [60].

Therefore, in the present study, we selected the expression levels of *IL-6* and *IL-1β* mRNA, as well as the content of NO, to preliminarily assess whether Ureidopropionic acid is involved in the regulation of inflammation following LPS stimulation of RAW264.7 cells. Additionally, we conducted molecular docking of nitric oxide synthase, a key target protein for NO production, with Ureidopropionic acid to predict its potential as a candidate for intervening in the cellular inflammatory system as one of the targets in the regulatory mechanism.

**Figure 10:**
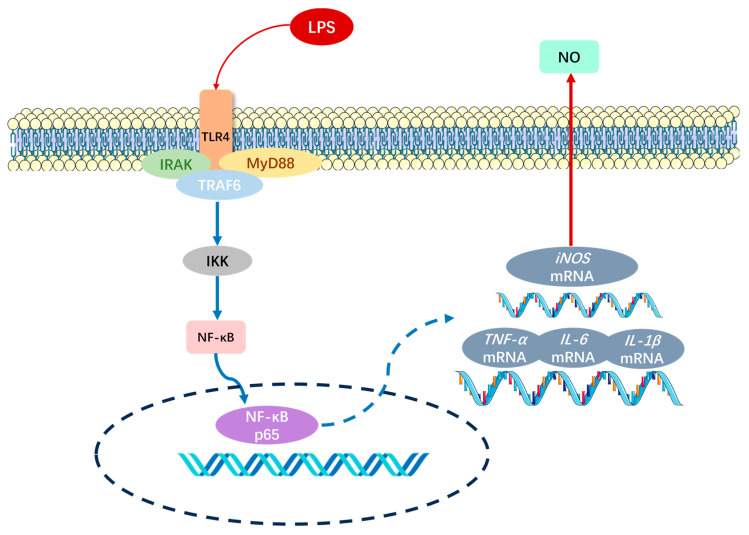
After lipopolysaccharide (LPS) stimulation of RAW264.7 cells, TLR4 responds to the stimulated immune response.

This research provides only a preliminary validation of the inflammatory regulatory role of Ureidopropionic acid. Further exploration of the underlying mechanisms by which Ureidopropionic acid participates in inflammatory regulation through nitric oxide synthase is warranted (Figure 10).

## 4. Materials and Methods

### 4.1. Retrieval Strategy and Screening Extraction of Literature Omics Data

Utilization of the PubMed database (https://pubmed.ncbi.nlm.nih.gov/ accessed on 12 September 2024), the retrieval terms included ‘RAW264.7, LPS, transcriptomics’, ‘RAW264.7, LPS, proteomics’, ‘RAW264.7, LPS, metabolomics’ with a search date from 1 January 2014 to 1 January 2024, collected relevant omics literature targeting LPS-induced RAW264.7 mouse macrophages. The heat map or Supplementary Table Data of the relevant omics in the literature were screened and extracted, respectively. For the data of screened genes, proteins, and metabolites, data collection, and extraction included omics classification, LPS intervention time point, differential genes or proteins or metabolites, and the above contents were classified and sorted. The origin of our searches was fixed to the ‘LPS, RAW264.7’ model, and all textual data collected in this baseline were collected, but data on differential expression were not included in our screening criteria.

### 4.2. Preprocessed of Original Omics Data

In our work, the original omics data of each group were preprocessed by manual normalization, and the processed omics data were classified into several gene ID matrices.

#### 4.2.1. Differential Genes of Transcriptomics

Used the Biomart database (http://asia.ensembl.org/biomart/martview/ accessed on 12 September 2024), Bioconductor version: Release (3.18). DataSet with Ensemble Genes-Mouse Gene selected the Input external references ID list-UniProtKB Gene Name in the Filters column, exported the Excel file, and the title bar which names NCBI gene (formerly Entrez Gene) ID was the gene ID obtained by batch conversion. For genes that cannot be converted in batches, the conversion of mouse gene ID was performed by querying the conversion separately in the UniProt database (https://www.uniprot.org/ accessed on 12 September 2024), Release 2024_02, GeneCards database (https://www.genecards.org/ accessed on 12 September 2024), Version 5.20 and NCBI database (https://www.ncbi.nlm.nih.gov/ accessed on 12 September 2024).

#### 4.2.2. Differential Proteins in Proteomics

The protein heat map or Supplementary Table Data of proteomics were used to query the gene encoding the protein of the mouse species through the UniProt database and then further converted into the gene Entrez Gene ID in the Biomart and NCBI databases.

#### 4.2.3. Metabolites of Metabolomics

Used the method of querying the related catalytic enzymes involved in the synthesis of metabolites in the KEGG database (https://www.genome.jp/kegg/ accessed on 12 September 2024), Release 110.0, and the related catalytic enzymes that used metabolites as substrates to produce new substances. Then UniProt, GeneCards, and NCBI were utilized to screen the genes of mice by using the enzymes obtained from the query, and they were also converted into Entrez Gene ID by batch conversion or single conversion.

### 4.3. Construction of Classification Sampling Model

After eliminating duplicates, the data normalized to Entrez Gene ID were put into the same matrix as the total set T. A subset M was selected from the total set T, and a total of N times was selected according to the time sequence. Subsequent biographical analysis was performed on each subset, respectively.

### 4.4. KEGG Pathway Enrich Analysis

The multi-gene pathway enrichment analysis was performed through the DAVID database (https://david.ncifcrf.gov/ accessed on 12 September 2024), DAVID Knowledgebase v2024q1 release, according to time resolution.

### 4.5. Pathway Intersection Analysis and Target Prediction

According to the results of multi-gene pathway enrichment at each time point, the Bioinformatics website (https://www.bioinformatics.com.cn/ accessed on 12 September 2024) was used to draw the Venn diagram for intersection analysis. Subsequently, they were output as mutual pathways and differential pathways with differential genes at each time point. After extracting the differential pathway with the smallest *p*-value and enriching the main substances in the differential pathway, the SwissTargetPrediction target prediction online platform (http://www.swisstargetprediction.ch accessed on 12 September 2024) was used to predict the potential active target of the substance.

### 4.6. Molecular Docking

The crystallographic texture of the target was retrieved and downloaded from the Protein Data Bank (PDB) database (http://www.rcsb.org/ accessed on 12 September 2024) at a resolution of less than 3 Å. The mouse source crystallographic texture was selected and saved in PDB format. The original ligand was removed from the protein crystallographic texture using the PyMOL software (PyMOL 2.5) (https://pymol.org/2/ accessed on 12 September 2024) and saved as a PDB file. The AutoDockTools software was used to remove water molecules, add polar hydrogen atoms, and assign charges to the protein. The 3D structure of the chemical composition was obtained from the PubChem database and saved as an SDF file, which was then converted to PDB format by the PyMOL software. In the AutoDockTools software, the ligands were processed by adjusting the charge, determining the root of the ligand, and selecting the twisted bond of the ligand before being saved in PDBQT format. The grid box module in the AutoDockTools software was used to set the X, Y, and Z coordinates to identify the active sites of the protein and generate a docking information file. Molecular docking was carried out in AutoDock Vina to determine the docking affinity (kcal/mol) of the active ingredient with the key target, with each docking process repeated three times. The affinity score was used to assess the binding of active ingredients to the receptors. Finally, the docking results were visualized using the PyMOL software.

### 4.7. Cell Culture and Activity Determination

Mouse monocyte-macrophage leukemia cell line RAW264.7 (Cell Bank, Chinese Academy of Sciences, Shanghai, China, TCM13) was cultured in high glucose DMEM medium (Vina Cell Biotechnology Co., Ltd. Shanghai, China, Lot. 2351429) containing 15% fetal bovine serum (Cellmax, FBS, Beijing, China) and a 1% penicillin–streptomycin mixture (Boster Bioengineering Co., Ltd., Wuhan, China) at 37 °C and 5% CO_2_ saturated humidity. RAW264.7 cells in the logarithmic growth phase were seeded in 96-well plates at a density of 1.5 × 10^5^ cells/mL. The cells were stimulated with 0.1 μg/mL LPS for 6 h and then cultured with 50, 25, 12.5, 6.25, 3.125, 1.5625, and 0.78125 μmol Ureidopropionic acid for 6 h, respectively. The cell viability was measured by CCK8 (Baoguang Biological, Chongqing, China, Lot. # X2544481X), and the absorbance value (OD value) was measured by Thermo Electron Corporation (Waltham, MA, USA) at 450 mm.

### 4.8. Determination of the Nitric Oxide (NO) Level

RAW264.7 cells in the logarithmic growth phase were seeded in 24-well plates at a density of 1 × 10^6^ cells/mL overnight and stimulated with 0.1 μg/mL LPS. After 6 h, the upper medium was discarded and treated with two concentrations of Ureidopropionic acid (6.25, 0.78125 μmol) for another 6 h. After the treatment, the cell culture medium was collected, and the supernatant was taken for NO level detection by the Griess method (A NO kit was purchased from Beyotime Biotechnology Co., Ltd., Shanghai, China, S0021S, Lot No. 052223231108).

### 4.9. Real-Time Fluorescence Quantitative PCR

RAW264.7 cells in the logarithmic growth phase were seeded in 24-well plates at a density of 1 × 10^6^ cells/mL overnight and stimulated with 0.1 μg/mL LPS. After 6 h, the upper medium was discarded and treated with two concentrations of Ureidopropionic acid (6.25, 0.78125 μmol) for 6 h.

RAW264.7 cells in the logarithmic growth phase were seeded in 24-well plates at a density of 1 × 10^6^ cells/mL overnight and stimulated with 0.1 μg/mL LPS. After 0.5 h, the upper medium was discarded and treated with seven concentrations of Ureidopropionic acid (25, 12.5, 6.25, 3.125, 1.5625, 0.78125, 0.390625 μmol) for 0.5 h.

After the treatment, the bottom cells were collected, and the RNA extraction kit (Lot. No. R230501) was used to extract the RNA from the cells. After the RNA concentration was measured and quantified, according to the reverse transcription kit (Servicebio Biotechnology Co., Ltd., Wuhan, China, Cat: G3337-100; lot. MPC2311007). The RNA was reverse transcribed into cDNA, and fluorescence quantification was performed using SYBR Prime (purchased from Baoguang Biological, Chongqing, China, Lot. # L1445255T). The mRNA expression levels of *IL-6*, *IL-1β,* and *TNF-α* were standardized by *β*-actin as an internal reference gene. The data were calculated by the 2^ΔΔCt^ method. The primer sequences used are as follows (Table 2):

### 4.10. Statistical Analysis

The data were processed by the GraphPad Prism 9.3.0 software, and the data were expressed as Mean ± SEM. The mean comparison between multiple groups was analyzed by the analysis of variance, and the pairwise comparison between multiple groups was performed using the *t* test. *p* < 0.05 was set as statistically significant.

## 5. Summary and Conclusions

This research utilized ensemble learning techniques to analyze a dataset related to omics literature on cell inflammation. By integrating and classifying the model, the study re-evaluated the pathway linked to β-alanine metabolism in RAW264.7 cells exposed to lipopolysaccharide (LPS) stimulation, a pathway that may have been previously neglected in the study of inflammatory systems. Furthermore, a novel anti-inflammatory compound, Ureidopropionic acid, was identified within the β-alanine metabolism pathway. An initial in vitro pharmacodynamic evaluation of Ureidopropionic acid demonstrated a dose–response relationship, particularly at lower concentrations, which significantly affected the expression levels of *TNF-α* mRNA. However, the lack of pharmacokinetic data has obscured the specific mechanism of action of this compound. Therefore, additional in vivo studies, the formulation of well-structured dosing regimens, and the implementation of techniques such as Ligand fishing and Surface Plasmon Resonance are imperative to ascertain whether Ureidopropionic acid acts as a single-target or multi-target therapeutic agent in future research endeavors.

## Figures and Tables

**Table 1:** Molecular docking binding energy results of Ureidopropionic acid with *Upb1*, *iNOS* encoded proteins, and the result of β-alanine with *Upb1*.

Chemical Compound	Protein	Gene	Affinity (kcal/mol)
UREIDOPROPIONIC ACID	Nitric oxide synthase	*iNOS*	−5.3
	Beta-ureidopropionase	*Upb1*	−5.1
β-ALANINE			−3.7

**Table 2:** The primer sequences in this work.

Gene	Gene Accession No.	Primer Sequence (5′–3′)
*IL-6*	NM_031168	F: CAAAGCCAGAGTCCTTCAGAG R: AGCATTGGAAATTGGGGTAG
*IL-1β*	NM_008361	F: TGGCAACTGTTCCTG R: GGAAGCAGCCCTTCATCTTT
*TNF-α*	NM_013693	F: CCCTCACACTCAGATCATCTTCT R: GCTACGACGTGGGCTACAG
*β-Actin*	NM_007393	F: TGACGGGGTCACCCACACTG R: AAGCTGTAGCCGCGCTCGGT

## Data Availability

The original contributions presented in the study are included in the article/Supplementary Materials; further inquiries can be directed to the corresponding authors.

## Supplementary Materials

The following supporting information can be downloaded at: https://www.mdpi.com/article/10.3390/ijms251910252/s1.
